# Continued Elevation of Plasma IL-4 and IL-17 Predicts the Progression from VMC to DCM

**DOI:** 10.1155/2020/9385472

**Published:** 2020-01-08

**Authors:** Z. H. Wang, Y. H. Liao, J. Yuan, X. J. Jin, M. Yu, R. Z. Chen, D. J. Xu, J. Wei, J. Wan, D. C. Zhao, H. Y. Han, B. Li, G. Tian, G. Hu, J. Xu

**Affiliations:** ^1^Department of Cardiology, Union Hospital, Tongji Medical College, Huazhong University of Science and Technology, Wuhan, China; ^2^Department of Cardiology, Fudan University, Zhongshan Hospital, Shanghai, China; ^3^Department of Cardiology, The First Affiliated Hospital of Nanjing Medical University, Nanjing, China; ^4^Department of Cardiology, The Second Affiliated Hospital of Xi'an Jiaotong University, Xi'an, China; ^5^Department of Cardiology, Zhongnan Hospital of Wuhan University, Wuhan, China; ^6^Department of Cardiology, The First Affiliated Hospital of Harbin Medical University, Harbin, China; ^7^Department of Cardiology, Tianyou Hospital Affiliated to Wuhan University of Science & Technology, Wuhan, China; ^8^Department of Cardiology, The Second Hospital of Shanxi Medical University, Taiyuan 030001, China; ^9^Department of Cardiology, First Affiliated Hospital Xi'an Jiaotong University, Xi'an, Shanxi, China; ^10^Department of Cardiology, Jingzhou Central Hospital, Jingzhou, Hubei, China; ^11^Department of Cardiology, Renji Hospital, Shanghai Jiaotong University School of Medicine, Shanghai, China

## Abstract

**Objective:**

To investigate plasma cytokines (interferon gamma, interleukin-4, and interleukin-17) in patients with viral myocarditis (VMC) and evaluate their predictive value in the progression from VMC to dilated cardiomyopathy (DCM).

**Methods:**

A prospective, multicenter, observational study included 536 patients with newly diagnosed VMC admitted in cardiology departments of 24 tertiary super specialised university-affiliated hospitals in the China registry from January 2012 to June 2016. Demographics and clinical characteristics at baseline and after three months were collected, including laboratory blood tests, ECG, echocardiography, and drug treatment in each participating site. The plasma anti-viral antibodies (Abs), anti-heart autoimmune Abs, and cytokines were detected by ELISA.

**Results:**

Of the 536 patients, 534 were included for analysis after two patients died in less than a month. The plasma levels of IFN-*γ*, IL-4, and IL-17 were continually higher in patients with incident DCM than in those without incident DCM at baseline, from the 1st month and the 3rd month; all had a *P* value of <0.0001. There was a positive correlation between IL-4 and LVEDd (*r* = 0.30, *P* < 0.0001) and between IL-17 and LVEDd (*r* = 0.11, *P* = 0.02). When all these covariates have entered the model simultaneously, elevated IL-4 and IL-17 were still significantly associated with DCM incidence. The RR (95% CI) of DCM incidence were 1.04 (1.02-1.06) for IL-4 and 5.24 (2.81-9.79) for IL-17.

**Conclusion:**

The continued elevation of plasma IL-4 and IL-17 in VMC patients were associated with a high incidence of DCM at three months, and these two cytokines were independent predictors for the progression from VMC to DCM.

## 1. Introduction

Viral myocarditis (VMC) is a common illness worldwide that can lead to severe complications or death in infants and young adults [1, 2], which is responsible for sudden death cases in young adults (8.6%-12%) and 9% to 16% of newly onset dilated cardiomyopathy (DCM) [3, 4]. The incidence of myocarditis is approximately 1.5 million cases worldwide per year [5]. Acute VMC, a precursor of DCM leading to heart failure, is a triphasic disease involving an initial viral infection, followed by autoimmune response, and finally remodelling of cardiac structure and function [6].

The pathogenesis of DCM secondary to VMC is closely associated with dysfunction of the autoimmune system. CD4^+^ Th cell subsets (Th1, Th17, and Th2) are involved in the mechanisms for the onset of VMC and DCM and the progressing from VMC to DCM [6–8]. The acute viral infection will activate the Th0 cells, and in so doing, this will initiate a cascade of events as follows: Th0 will differentiate to Th1 cells resulting in the production of IFN-*γ*, thus fueling myocardial cell injury. In the acute stage, Th0 will also differentiate to Th17 cells resulting in the production of IL-17. IL-17, on the one hand, works on cytotoxic T lymphocytes (CTLs) to lead to myocardial cell injury, and on the other hand, it also works on B lymphocytes to produce several anti-heart autoantibodies (AHAs). In the chronic stage, Th0 differentiates to Th2 cells causing the production of IL-4, which promotes B cells to produce AHAs. AHAs can mediate Ca^2+^ influx into the myocardial cells and damage them [6–12]. The objective of our study was to observe the transforming rate progressing from VMC to DCM in China and to decide if cytokines (IL-17, IL-4, and IFN-*γ*) could predict this progression.

## 2. Methods

### 2.1. Study Population

A total of 536 patients with newly diagnosed clinical VMC admitted in 24 cardiology departments of tertiary super specialised hospitals in China were enrolled in the registry in each participating center from January 2012 to June 2016. There were no specific exclusion criteria. A signed informed consent was obtained.

### 2.2. Study Design

This is a prospective, multicenter, observational study. Demographics and clinical characteristics at baseline and after three months were collected from all patients, including laboratory blood tests, ECG, echocardiography, and drug treatment at each participating site. The plasma anti-viral antibodies (Abs), anti-heart autoimmune Abs, and cytokines were detected by ELISA. VMC clinical types enrolled for the study were divided by four categories: arrhythmia type, heart failure type, acute severe type, and subclinical type according to the 1999 Chinese viral myocarditis diagnostic standard and the 2013 ESCs [4, 13]. The following VMC clinical types were enrolled for the study:

- Arrhythmia type: 1 to 3 weeks after virus infection, mild precordial discomfort, palpitations, ECG reveals premature beats or tachycardia, atrioventricular block, and ST-T changes. There was increased TnI, no clinical manifestations of heart enlargement and heart failure, and gradual recovery after 1 to 2 months of treatment
- Heart failure type: 1-3 weeks after the virus infection, fatigue, palpitation, dyspnea and other symptoms, increased TnI, cardiac enlargement and heart failure can be associated with arrhythmia, and some patients evolved to dilated cardiomyopathy
- Acute severe type: 1 to 2 weeks after the virus infection, chest pain, palpitation, difficulty in breathing, occurrence of ventricular tachycardia, ventricular gallop, heart failure, cardiac enlargement and other clinical manifestations or even cardiogenic shock, with TnI significantly increased. This type of illness is dangerous, and some patients showed eruption of myocarditis and could have died within a few days or weeks due to pump failure or severe arrhythmia
- Subclinical type: no symptoms after virus infection and ECG detected ST-T changes which may disappear early in the morning or a few weeks later

The definition for VMC progressing to DCM is the appearance or persistence of LVEDd ≥ 5.5 cm and LVEF < 45% in patients given optimal medical treatment and followed up for three months [13].

### 2.3. Statistical Analysis

Baseline characteristics of the viral myocarditis patient cohort were summarised according to the incidence of dilated cardiomyopathy for three months follow-up. All data are presented as mean ± SD for continuous variables, median (interquartile range) if continuous variables were skewed, and *n* (%) for categorical variables. Comparisons between groups were performed by the chi-square or Fisher exact test (for categorical variables) and the Student *t*-test or Mann-Whitney *U* test (for continuous variables). Linear mixed-effect models and logistic regression models with adjustment for gender, age, and baseline echocardiography covariables were used to identify the change of cytokine status (after natural logarithmic transformation) that pose the highest risk for incident DCM. The C statistics for each risk factor were calculated to estimate the predictive values of incident DCM. A *P* value of <0.05 was considered statistically significant. All statistical analyses were performed using SAS version 9.3 (SAS Institute Inc., Cary, NC).

## 3. Results

### 3.1. Patient Characteristics and Incident DCM

Included in the study were 536 patients of a VMC cohort, of which two patients died in less than one month. A comparison of baseline clinical and laboratory parameters between patients with and without DCM is presented in Table 1. After three months follow-up, 127 (23.78%) newly onset DCM were recorded among 534 patients of the VMC cohort; out of the 127 patients who reached the primary endpoint, significant types were in 46 patients (36.22%) of the acute severe type and in 62 patients (48.82%) of the heart failure type. In comparison with patients without DCM, those patients with DCM presented at an older age, with lower LVEF, larger LVEDD, worse NYHA class, higher NT-pro-BNP levels, and an increased virus infection rate (all with *P* < 0.0001). Baseline antibodies (against ANT, *β*1R, MHC, and CaC) and cytokines (IFN-*γ*, IL-4, and IL-17) were significantly higher in patients who developed DCM as compared to those who did not develop DCM.

### 3.2. Prediction of Incident DCM in Patients with VMC

**Figure 1:**
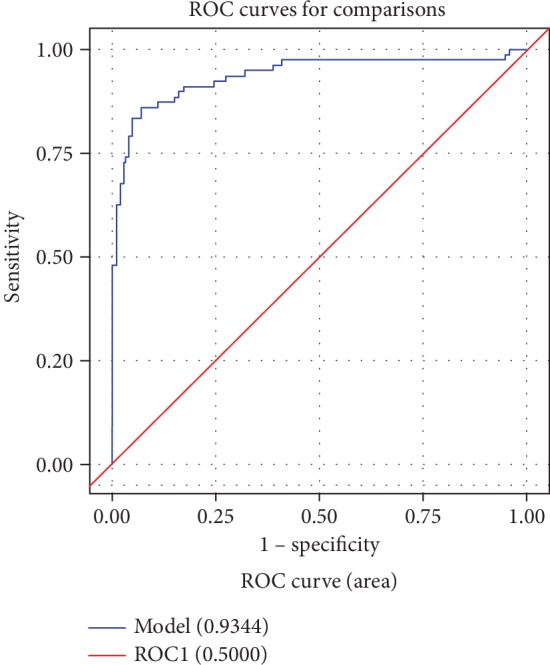
Predicted 3-month risk of incident DCM, age, gender, and LVEF+ln(IL-4)+ln(IL-17).

Univariate analysis shows all variables concerning incident DCM after three months (Table 2). Adjusted relative risks of incident DCM after three months, multivariate analysis of age, LVEF, IL-4, and IL-17 predicted the occurrence of DCM; the RR (95% CI) were between 0.895 (.866-0.925) and 5.24 (2.81-9.79). Patients with elevated levels of IL-4 and IL-17 have a high risk of developing DCM in patients with VMC; on the addition of IL-4 and IL-17 to the standard model (age, sex, and LVEF), the predictive power of progression to DCM is improved. For the prediction of incident DCM in patients with VMC (Table 3 and Figure 1), IL-4 and IL-17 significantly enhanced the area under the ROC curve (AUC) in the predicted 3-month risk of DCM vs. the basic demographic model (age, sex, and LVEF). VMC patients with higher levels of IL-4 and IL-17 have a higher risk of developing DCM. On the addition of ln(IL-4) and ln(IL-17) to the standard model (age, sex, and LVEF), the corrected area under the curve increased from 0.9155 (95% CI, 0.8769-0.9540) to 0.9344 (95% CI, 0.8948-0.9741) for predicting the incidence of DCM events.

### 3.3. Correlation between Dynamic Changes of Cytokines and Occurrence of DCM

**Figure 2:**
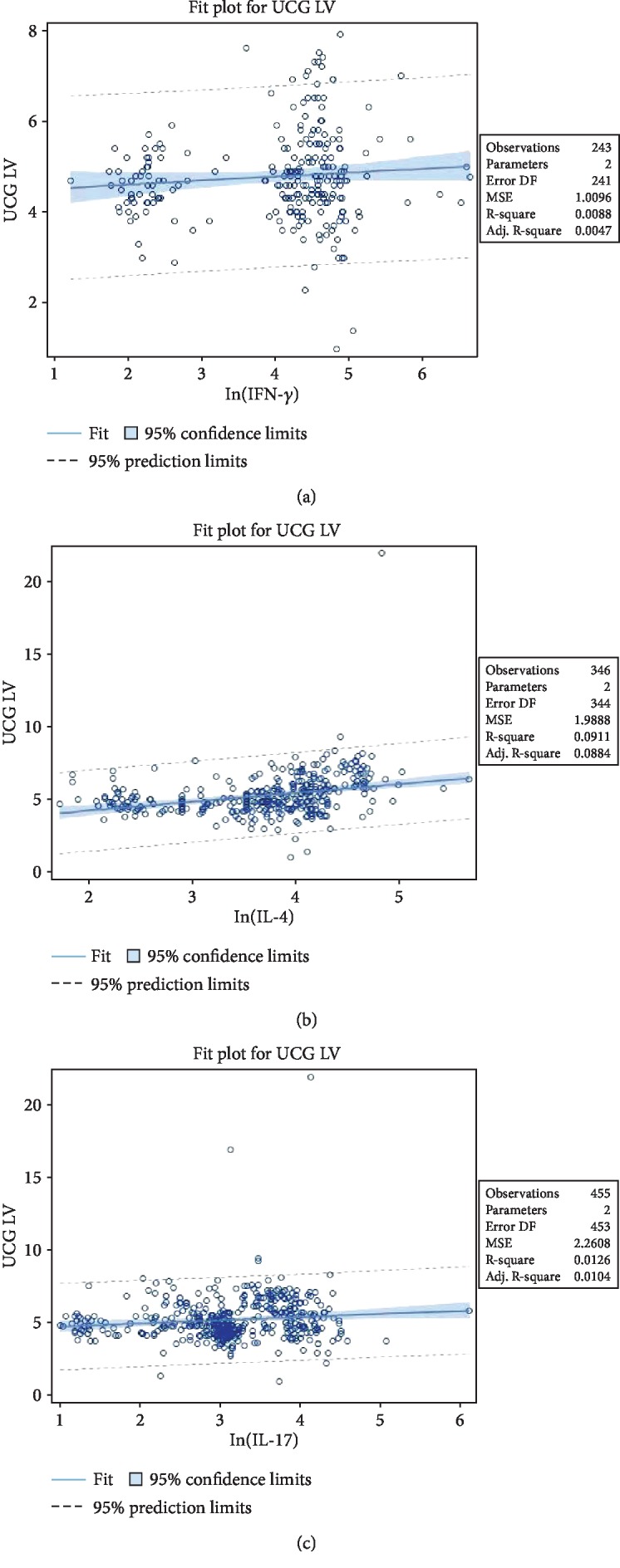
Correlation between baseline echocardiographic parameters (LV) and IFN-*γ*, IL-4, and IL-17. (a) No relationship was seen between baseline LV and ln(IFN-*γ*), *r* = 0.09380, *P* = 0.1449. (b and c) IL-4 and IL-17 showed similar significant positive correlations with UCG LV, with the highest for IL-4 (correlation coefficient *r* = 0.30177, *P* ≤ .0001) and IL-17 (correlation coefficient *r* = 0.11218, *P* = 0.0167).

The dynamic changes of cytokines and the appearance of DCM, in which patients with VMC who presented with high levels of IFN-*γ*, IL-4, and IL-17 had a higher risk of developing DCM, are summarised in Table 4. Dynamic observation showed that the levels of plasma IFN-*γ*, IL-4, and IL-17 were continually higher in patients with incident DCM than in those without DCM at baseline, the 1st month, and the 3rd month (all *P* < 0.0001) as shown in the fit plot in Figure 2. For the relationship between cytokine (IFN-*γ*, IL-4, and IL-17) levels and echocardiographic parameters (LV), after adjusting for age, gender, and LVEF as well as NT-pro-BNP, IL-4 and IL-17 showed similar significant positive correlations with UCG LV, and the highest Pearson correlation was in IL-4 (correlation coefficient *r* = 0.30177, *P* = <.0001) followed by IL-17 (correlation coefficient *r* = 0.11218, *P* = 0.0167). The mean LV size for patients with DCM was 6.7 ± 2.2 cm, and the mean LVEF was 34.8 ± 9.7%, which were all statistically significant with the *P* value of <0.0001. There was no relationship between baseline LV and ln(IFN-*γ*), *r* = 0.09380, *P* = 0.1449. Therefore, the continued elevation of IL-4 and IL-17 can predict the incidence of DCM.

## 4. Discussion

The present study portrays the incidence of dilated cardiomyopathy from 534 patients who were diagnosed with having clinical viral myocarditis during a mean follow-up of three months, during which 127 (23.78%) patients reached the primary endpoint (incident DCM); several studies revealed different percentages implicating myocarditis of newly onset dilated cardiomyopathy cases [3, 4, 14]. Here, we first achieved the acquisition of Chinese data about the transforming rate, which gave us a clear picture from the Chinese registry perspective.

Moreover, we further explored the role of cytokines in the progression of myocarditis to develop dilated cardiomyopathy. Although it was found that Th cells and related cytokines exert an important function in the progress of VMC to DCM, the cytokines' clinical significance as predictors of incident DCM was scantly investigated. Through a multivariate model, four variables were found to be associated with the progression to DCM, and these were age, LVEF, IL-4, and IL-17. On further analysis, IL-4 and IL-17 significantly improved the area under the ROC curve (AUC) in predicting the 3-month risk of DCM vs. the basic demographic model (age, sex, and LVEF). Increased levels of IFN-*γ* in the current study did not present any significant function in predicting progression from VMC to DCM. The dual role of IFN-*γ* can be in VMC pathogenesis, wherein in one hand, when increased it mediates myocardial cell injury, and on the other hand, it inhibits viral replication.

Furthermore, 15 patients out of 99 with subclinical types of VMC were observed progressing to DCM, which was expected in the advanced stages of the disease. A key point to be emphasised is that in developing DCM, the autoimmune mechanism plays a substantial role and not the severity of the patient's baseline condition. So, patients of this type require attention if IL-4 and IL-17 levels are high even when their symptoms are mild.

The baseline UCG left ventricular dimension could not predict the incident DCM. Then, we assessed the correlation between LV dimension and cytokines, where we found positive associations between IL-4 or IL-17 and UCG LV dimension. Despite the above fact, there was no correlation between IFN-*γ* and UCG LV dimension. Both dynamic changes of IL-4, IL-17, and UCG LV dimension correlations indicated that continued elevation of plasma IL-4 and IL-17 could predict the progression from VMC to DCM.

## 5. Limitations

The present study is based on a multicenter observational registry of VMC patients. The critical limitation of this registry is that it lacks endomyocardial biopsy because of the nature of the test as an invasive clinical tool, and it is challenging for patients to accept in China.

## 6. Conclusion

The rate of transformation from VMC to DCM was about 23.78% in China, which was in concordance with other observational studies from other countries. The continued elevation of IL-4 and IL-17 in VMC patients was associated with a high incidence of DCM. Since detection for plasma IL-4 and IL-17 is not that complicated, they may serve as significant predictors for VMC evolving to DCM.

## 7. Key Points

*Question.* What is the role of cytokines (IL-4, IL-17, and IFN-*γ*) in predicting progression from VMC to DCM?

*Findings.* In this prospective multicenter observational study of 536 patients hospitalized with clinical VMC, elevated levels of plasma IL-4 and IL-17 were associated with a high incidence of DCM at 3 months, and these two cytokines were the independent predictors for the progression from VMC to DCM.

*Meaning.* Inflammation through inflammatory cytokines is essential for the progression of VMC to DCM. IL-4 and IL-17 are significant predictors of incident DCM.

## Figures and Tables

**Table 1:** Comparison of baseline clinical, morphometric, and laboratory parameters between without and with DCM according to treatment response.

	All patients	Subgroups		*P* value
	With follow-up (*n* = 534)	Without DCM (*n* = 407)	With DCM (*n* = 127)	
Age (yrs), mean ± SD	35.81 ± 15.19	32.0 ± 13.3	48.1 ± 14.5	<0.0001
Male (%)	296 (55.43%)	211 (51.84%)	85 (66.93%)	0.0028
VMC subtype				<0.0001
Acute severe type	81 (15.17%)	35 (8.6%)	46 (36.22%)	
Heart failure type	139 (26.03%)	77 (18.92%)	62 (48.82%)	
Arrhythmia type	215 (40.26%)	211 (51.84%)	4 (3.15%)	
Subclinical type	99 (18.54%)	84 (20.64%)	15 (11.81%)	
NYHA III–IV	145 (27.90%)	70 (17.20%)	79 (62.2%)	<0.0001
ECG with arrhythmia	418 (78.28%)	303 (74.20%)	116 (91.3%)	<0.0001
ECG with ST-T changes	252 (47.01%)	153 (37.41%)	99 (77.95%)	<0.0001
Echocardiogram				
Left atrium (cm)	3.6 ± 0.84	3.3 ± 0.6	4.6 ± 0.8	<0.0001
Right atrium (cm)	3.65 ± 0.88	3.4 ± 0.7	4.5 ± 1.0	<0.0001
Left ventricle (cm)	5.27 ± 1.52	4.8 ± 0.8	6.7 ± 2.2	<0.0001
Right ventricle (cm)	2.96 ± 0.92	2.9 ± 0.9	3.1 ± 1.1	0.1946
LVEF (%)	52.61 ± 15.14	58.0 ± 12.0	34.8 ± 9.7	<0.0001
*E*/*A* < 1	110 (30.47%)	88 (29.04%)	22 (37.93%)	0.156
Laboratory				
WBC (G/L)	7.47 ± 2.76	7.5 ± 2.9	7.5 ± 2.3	0.9646
ALT (U/L)	26.5 (16-48.05)	26.0 (15.0-43.0)	32.6 (19.0-68.0)	0.0122
Cr (*μ*mol/L)	71.7 (57.3-86.2)	70.0 (56.0-82.3)	80.0 (65.3-99.0)	<0.0001
Coxsackievirus B5-IgM	357 (66.85%)	247 (60.69%)	110 (86.61%)	<0.0001
Coxsackievirus B3-IgM	344 (64.42%)	230 (56.51%)	114 (89.76%)	<0.0001
Cytomegalovirus-IgM	269 (50.37%)	166 (40.79%)	103 (81.1%)	<0.0001
Enterovirus RNA	351 (65.73%)	238 (58.48%)	113 (88.98%)	<0.0001
Anti-ANT antibody	433 (81.09%)	311 (76.41%)	122 (96.06%)	<0.0001
Anti-MHC antibody	224 (41.95%)	135 (33.17%)	89 (70.08%)	<0.0001
Anti-*β*1R antibody	287 (53.75%)	196 (48.16%)	91 (71.65%)	<0.0001
Anti-CaC antibody	263 (49.25%)	172 (42.26%)	91 (71.65%)	<0.0001
CK-MB (ng/mL)	13.8 (3.9-28)	14 (4-30)	13 (1.7-17)	0.1013
TNI-T (ng/mL)	0.05 (0-0.9)	0.05 (0-1.03)	0.02 (0.01-0.16)	0.4629
NT-pro-BNP (pg/mL)	300 (55.8-2186)	189.9 (35.0-1691.0)	1462.3 (229.0-3042.0)	<0.0001
IFN-*γ* (pg/mL)	79.24 (12.99-106.33)	76.48 (11.00 -105.56)	96.95 (80.70 -122.57)	0.0165
IL-4 (pg/mL)	45.29 (28.14-64.11)	38.24 (20.91 -55.98)	84.25 (59.20 -103.74)	<0.0001
IL-17 (pg/mL)	21.19 (14.73-41.88)	19.44 (12.68 -25.11)	38.81 (30.42 -50.42)	<0.0001
Medications				
*Astragalus membranaceus*	213 (39.89%)	162 (39.80%)	51 (40.16%)	0.9433
CoQ10	296 (55.43%)	229 (56.27%)	67 (52.76%)	0.4873
Vitamin C	205 (38.46%)	162 (39.90%)	43 (33.86%)	0.2218
ACEIs/ARBs	114 (21.35%)	87 (21.38%)	27 (21.26%)	0.9778
Beta blocker	218 (40.82%)	175 (43.00%)	43 (33.86%)	0.0673
Amiodarone	28 (5.24%)	20 (4.91%)	8 (6.30%)	0.5409

Means ± standard deviations and proportions of characteristics at baseline were estimated by incident DCM status. With DCM = incident of dilated cardiomyopathy, compared to without DCM.

**Table 2:** Adjusted relative risks (95% confidence interval) of incident DCM after three months.

Baseline characteristics	Univariate model^‡^	Multivariate model^§^
Age (yrs)	1.08 (1.06-1.10)^†^	1.036 (1.008-1.066), *P* = 0.0002^∗^
Male	1.88 (1.24-2.86)^†^	NS
LVEF (%)	0.88 (0.86-0.90)^†^	0.895 (0.866-0.925), *P* < 0.0001^†^
Ln(IFN-*γ*)	1.85 (1.14-2.98)^∗^	NS
Ln(IL-4)	1.06 (1.05-1.07)^†^	1.039 (1.022-1.055), *P* < 0.0001^†^
Ln(IL-17)	3.65 (2.56-5.20)^†^	5.241 (2.806-9.789), *P* < 0.0001^†^
Ln(NT-pro-BNP)	1.236 (1.102-1.386)^†^	NS
Coxsackievirus B5-IgM (+)	4.19 (2.42-7.25)^†^	NS
Coxsackievirus B3-IgM (+)	6.75 (3.68-12.37)^†^	NS
Cytomegalovirus-IgM (+)	6.23 (3.83-10.13)^†^	NS
Enterovirus RNA (+)	5.73 (3.18-10.33)^†^	NS
Anti-ANT antibody (+)	7.53 (2.99-18.96)^†^	NS
Anti-*β*1R antibody (+)	4.72 (3.06-7.27)^†^	NS
Anti-MHC antibody (+)	2.72 (1.77-4.19)^†^	NS
Anti-CaC antibody (+)	3.45 (2.24-5.33)^†^	NS

Ln: natural logarithmic transformation. ^‡^Model 1: univariate model. ^§^Model 2: multivariate model, all variables were entered into the model. ^†^*P* < 0.001. ^∗^*P* < 0.05.

**Table 3:** Prediction of incident DCM in patients with VMC (RR 95% CI).

	AUC (95% CI)
Age (yrs)	0.7961 (0.7526-0.8395)^†^
Gender	0.5767 (0.5290-0.6244)^∗^
LVEF (%)	0.9092 (0.8822-0.9362)^†^
UCG LV	0.9075 (0.8689-0.9461)
Ln(IL-4)	0.8566 (0.8057-0.9075)^†^
Ln(IL-17)	0.7682 (0.7223-0.8141)
Basic model (without cytokine, age+gender+LVEF)	0.9155 (0.8769-0.9540)
Basic model+ln(IL-4)+ln(IL-17)	0.9344 (0.8948-0.9741)^†^

^†^ *P* < 0.001. ^∗^*P* < 0.05.

**Table 4:** Longitudinal change of IFN-*γ*, IL-4, and IL-17 (pg/ml) levels in VMC patients with the incident of DCM.

	Without DCM (*n* = 407)			With DCM (*n* = 127)			*P* value
	Median	1st quartile	3rd quartile	Median	1st quartile	3rd quartile	
IFN-*γ*							
Baseline	76.5	11.0	105.6	97.0	80.7	122.6	0.0165
1st month	79.4	18.8	116.8	137.4	109.7	161.8	<.0001
3rd month	77.6	12.3	115.0	173.9	126.5	204.7	<.0001
*P* for trend	0.1334			<.0001			
IL-4							
Baseline	38.35	21.05	56.57	84.25	59.2	103.74	<0.0001
Visit 1 (1st month)	34.04	19.1	55.35	92.3	64.85	113.65	<0.0001
Visit 2 (3rd month)	27.3	17.15	53.58	113.04	79.43	139.19	<0.0001
*P* for trend	0.5157			<0.0001			
IL-17							
Baseline	19.32	12.63	24.72	38.81	30.42	50.42	<0.0001
Visit 1 (1st month)	18.06	12.55	20.64	43.67	37.25	50.1	<0.0001
Visit 2 (3rd month)	16.9	12.67	19.16	51.68	44.08	59.27	<0.0001
*P* for trend	0.1348			0.0235			

Data are expressed as median (1st Quartile-3rd Quartile). *P* for trend means the mixed-effect model. Detecting three times for the sample deciding if there is longitudinal change or not.

## Data Availability

The deidentified participant data are accessible by contacting the corresponding author Yuhua Liao via liaoyh27@163.com.
